# Burn-Induced Organ Dysfunction: Vagus Nerve Stimulation Improves Cardiac Function

**Published:** 2010-06-21

**Authors:** Andreas D. Niederbichler, Stephan Papst, Leif Claassen, Andreas Jokuszies, Kyros Ipaktchi, Kerstin Reimers, Tobias Hirsch, Lars Steinstraesser, Theresia Kraft, Peter M. Vogt

**Affiliations:** ^a^Department of Plastic, Hand and Reconstructive Surgery, Medizinische Hochschule Hannover, Burn Center, Hannover, Germany; ^b^Center for Complex Fractures and Limb Restoration, Denver Health System, Denver, Colo; ^c^Department of Plastic and Reconstructive Surgery, Hand Surgery, University of Bochum, Germany; ^d^Department of Molecular and Cellular Physiology, Medizinische Hochschule Hannover, Hannover, Germany

## Abstract

**Introduction:** Many studies have demonstrated the existence of an anti-inflammatory, parasympathetic pathway, termed as the *inflammatory reflex*. Burn-induced heart failure has been investigated in many previous studies. Proinflammatory cytokines, such as TNF-α, IL-1β, and IL-6, have been shown to play a key pathogenetic role and vagus nerve stimulation attenuates proinflammatory cytokine production. This study was designed to evaluate postburn alterations of cardiac functional parameters after vagal electrostimulation. **Material and Methods:** A 30% total body surface area standardized, full-thickness rat burn model was used. Electric stimulation of the vagus nerve was performed. The following functional cardiac parameters were measured by ventricular microcatheterization: Maximal and minimal left ventricular pressure, mean left ventricular pressure, end-diastolic pressure (EDP), positive and negative pressure rise and fall (±d*P*/d*t*), cardiac contractility index, and assessment of the heart rate. **Results:** Vagus nerve stimulation improved maximal and minimal left ventricular pressure values compared with burn-only animals. End-diastolic pressure was elevated significantly in stimulated animals; however, EDP values were comparable with those in sham-injured animals. Analyzing positive and negative pressure development, ±d*P*/d*t* was restored to levels measured in sham-injured animals but not to control animal levels. No variations in heart rate were found. **Conclusion:** We as well as others have shown that inflammation after burn injury is a key pathogenetic element, and this study provides new evidence that cardiac function is also improved by vagus nerve stimulation. These results lead us to consider novel therapeutic options for the treatment of postburn cardiac dysfunction.

Within the last years, the multifactorial pathogenesis of burn-induced cardiac dysfunction has been experimentally evaluated. A couple of immunologic systems and mediators have been proven to play central roles in the pathogenesis of cardiac contractility deficits after thermal trauma. A parasympathetic, anti-inflammatory pathway, known as the *inflammatory reflex*, has been implicated to play a central role in various experimental models of inflammation.^1^,^2^ It is known that activation of the α7 nicotinic acetylcholine receptor subunit (α7nAChR) by physical and pharmacological means attenuates the generation of multiple proinflammatory mediators.^1^^-^3 On a cellular level, previous work by Borovikova and colleagues^1^ has shown that activation of the inflammatory reflex by acetylcholine exposure blocks the release of proinflammatory mediators by macrophages. Acetylcholine exposure of α7nAChR-deficient macrophages did not change their cytokine secretion profile. Various clinical studies including patients with inflammatory diseases such as chronic inflammatory bowel diseases have revealed beneficial effects of parasympathetic stimulation.^4^,^5^ Previous research efforts have demonstrated that burn injury leads to cardiac dysfunction, which has been attributed to functional rather than structural changes of the cardiomyocyte. Cardiodepressive mediators such as TNF-α, IL-1β, and IL-6 have been identified as key pathogenetic elements. Experimental data suggest that TNF-α, IL-1β, and IL-6 are primary cytokines that lead to the deterioration of cardiac function, either individually or in combination.^6^ We have recently shown in an experimental burn trauma model that electrical stimulation of the cervical portion of the vagus nerve induces a significant reduction of circulating levels of the cytokines TNF-α, IL-1β, and IL-6.^7^ Measuring the cytokine content of organ homogenates, analyses revealed markedly reduced levels after electrical vagus nerve stimulation on the organ level as well.^7^ However, in experimental burn models, it is unknown whether activation of the inflammatory reflex leads to improved cardiac function. In the present study, we hypothesized that activation of the parasympathetic, anti-inflammatory pathway improves burn-induced cardiac dysfunction. The present study has been designed to evaluate whether electrical cervical vagus nerve stimulation after burn injury leads to improved cardiac function.

## MATERIAL AND METHODS

### Reagents

Unless otherwise indicated, all reagents were purchased from Sigma Aldrich (Sigma Aldrich Chemie GmbH, Munich, Germany).

### Experimental animals

Adult male Sprague-Dawley rats (Harlan Winkelmann GmbH, Borchen, Germany), weighing 300 to 350 g, were used in all experiments. Prior to use, the animals were housed in a specific, pathogen-free environment and allowed to acclimate to their surroundings for 1 week. Standard rat chow and water were available to the animals ad libitum and the animals were kept at a 12-hour light-dark cycle. All experiments were performed in accordance with the guidelines set forth by the German State Department for Animal Protection and Welfare as well as the German Society for Experimental Animal Science and in accordance with Federal German Animal Protection Law (GV-SOLAS, TSchG). The experimental protocol was approved by the University Committee on Use and Care of Animals at the Medizinische Hochschule Hannover and the Lower Saxony State Board on the Care and Use of Animals (LAVES, Oldenburg, Germany).

### Burn procedure

The scald burn injury was inflicted as previously described by Steinstraesser et al.^8^ Under isoflurane anesthesia, dorsal hair was closely clipped and remainders removed using Veet depilatory cream (Reckitt Benckiser GmbH, Mannheim, Germany). The rats were then placed in a prefabricated mold device with a rectangular opening that exposed the dorsal skin surface while protecting the remainder skin from burn exposure. The exposed skin surface was immersed in warm water (60°C) for 40 seconds, producing a full-thickness dermal burn over 30% of the total body surface area (TBSA). Sham animals underwent an identical procedure, except that they were immersed in room temperature water (24°C). Control animals received no analgesia, no room temperature water immersion, and no depilation or fluid resuscitation. The rats were immediately dried and resuscitated with Ringer's lactate solution (4 mL/kg/ per % TBSA burn). One half of the calculated resuscitation volume was given intraperitoneally and the remaining volume was given as multiple doses by subcutaneous injections immediately postburn. The animals also received novamine sulfone-acid (ratiopharm GmbH, Ulm, Germany) for analgesia after induction of burn injury. Sham animals received the same resuscitation and analgesia treatment but no burn injury.

According to the experimental group (CTRL, control group; BURN, burn trauma BURN + STIM, burn trauma plus vagus nerve stimulation; and SHAM, sham group), animals received electrical stimulation of cervical vagus nerve trunks (pulsed electric vagus nerve stimulation for 12 minutes in parallel to the burn injury and 60-minute postinjury [5 V, 40-ms pulse duration, 1 Hz]). Three hours after burn or sham injury, all animals underwent left ventricular microcatheterization and assessment of cardiac functional parameters. Subsequently, animals were killed humanely according to the experimental protocol.

### Electrical stimulation of cervical vagus nerve trunks

Animals were kept under continuous isoflurane anesthesia and continuous analgesia postburn to carry out the vagus nerve stimulation procedure, which was initiated in parallel to the burn trauma. An anterior cervical approach was performed via midline vertical skin incision. After blunt preparation, both vagus nerve trunks were carefully freed from surrounding tissue and separated from the carotid artery trunks by using microsurgical techniques. Care was taken not to injure either the vagus nerve trunk or the carotid artery trunk. Subsequently, an insulating dry rubber sheet was slid under both vagus nerve trunks, and a custom-made bipolar platinum electrode clip system was used to ensure constant and safe flow of electrical current without electrical flow to the surrounding tissue (Research Laboratories of Hannover Medical School, Hannover, Germany). After the electrode system was brought in place, the vagus nerve trunks were cut proximally to ensure efferent stimulation. The system was connected to a Grass S-8 stimulator (Grass Instruments, Inc, Warwick, RI) and pulsed electric vagus nerve stimulation was performed for 12 minutes in parallel to the burn injury and 60 minutes postinjury (5 V, 40-ms pulse duration, 1 Hz) as described earlier.^7^

### Left ventricular microcatheterization and functional assessment

Rats were deeply anesthetized with isoflurane anesthesia at time *t* = 3 hours after induction of burn injury and anticoagulated with 1000 units intraperitoneal injection of heparin (Liquemin, Roche-Pharma AG, Grenzach-Wyhlen, Germany). The right carotid artery was exposed, and a 2.5-French microtip catheter (Millar Instruments, Houston, Tex) was advanced into the left ventricle. Correct positioning in the left ventricle was verified as seen in results. Maximal and minimal left ventricular pressure (LVP_max/min_, mm Hg), mean left ventricular pressure (LVP_mean_, mm Hg), end-diastolic pressure (EDP, mm Hg), positive and negative pressure rise and fall (±d*P*/d*t*, ±mm Hg/s), and the heart rate (HR, beats per minute, bpm) were recorded for a minimum of 5 minutes, using a signal transduction and amplification system connected to a standard Microsoft Windows operating system PC equipped with the appropriate recording and analysis software (PowerLab 8SP Base, Bridge Amp, Chart 5 Software, ADInstruments GmbH, Heidelberg, Germany).

### Statistical analysis

Results are expressed as the mean value ± standard error of the mean unless otherwise noted. Analysis of variance (ANOVA) followed by Tukey post hoc tests or Student *t* tests were used to test for differences among the experimental groups. Statistical significance was defined as *P* ≤ .05.

## RESULTS

### Maximal and minimal left ventricular pressure

**Figure 1:**
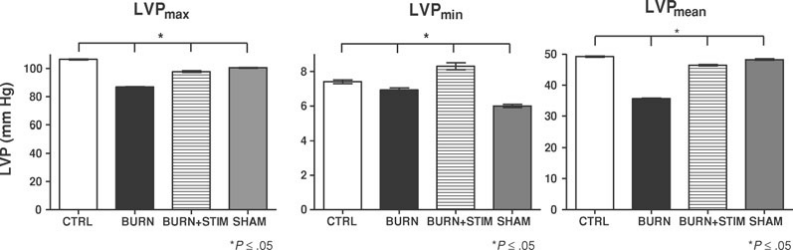
Analysis of minimal, maximal, and mean left ventricular pressure (LVP_max/min/mean_). Vagus nerve stimulation induced normalization of LVP_max_ values (*left panel*, BURN vs BURN + STIM bars), LVP_min_ levels were increased to levels above those found in control animals (*middle panel*, CTRL vs BURN + STIM bars). Sham animals displayed significantly decreased LVP_min_ levels compared with controls (*middle panel*, CTRL vs SHAM bars). Mean left ventricular pressure analysis (*right panel*, LVP_mean_). BURN group animals displayed a significantly lower mean LVP than their noninjured (*right panel*, CTRL and SHAM) counterparts (*right panel*, CTRL and SHAM vs BURN, white and gray bars vs black bar, *P* ≤ .05, analysis of variance). Vagus nerve electrostimulation induced restoration of normal mean LVP values (*right panel*, CTRL vs BURN + STIM, *P* = NS).

Maximal and minimal left ventricular pressure was measured and analyzed. Figure 1 depicts the results of the LVP experiments: mean LVP_max_ values were significantly elevated in animals that received burn injury and vagus nerve stimulation compared with animals with burn trauma alone (Fig 1, left panel). LVP_max_ levels did not completely recover back to normal (CTRL) levels with vagus nerve stimulation (Fig 1, left panel; CTRL bar vs BURN + STIM bar, *P* ≤ .05, ANOVA). Upon vagal stimulation, LVP_min_ levels were elevated to levels above the control group levels in the BURN + STIM group; however, this effect was not statistically significant (Fig 1, middle panel, CTRL vs BURN + STIM bar, *P* = NS, ANOVA).

### Mean left ventricular pressure

**Figure 2:**
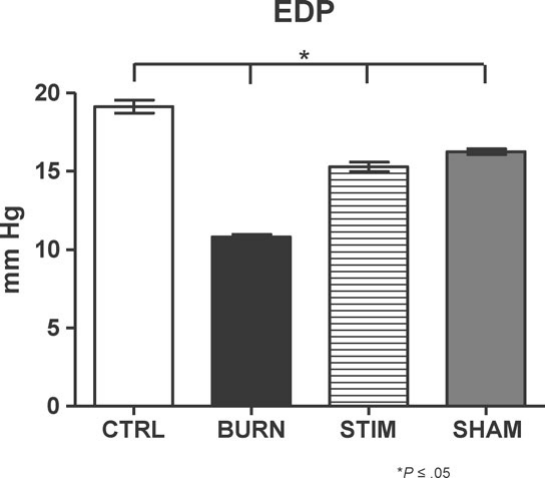
End-diastolic pressure (EDP) analysis. Sham animals had decreased EDP levels compared with controls. Burn injury plus vagus nerve stimulation induced restoration of the EDP levels but not normalization back to control levels after vagus nerve stimulation (SHAM vs CTRL groups [*P* ≤ .05]; STIM vs CTRL [*P* ≤ .05]; and SHAM [*P* = NS] groups).

Mean left ventricular pressure was analyzed in all experimental groups. Figure 2 displays the results of these experiments, control animals revealed LVP_mean_ levels ranging around 50 mm Hg, whereas burn-only animals showed a significant pressure drop to levels around 35 mm Hg (Fig 1, right panel; CTRL vs BURN groups, *P* ≤ .05). Burned animals that underwent posttraumatic vagus nerve stimulation displayed restoration of the LVP_mean_ values back to levels of their control and sham-injured counterparts (Fig 1, right panel; STIM vs CTRL and SHAM groups, *P* = NS).

### End-diastolic pressure

**Figure 3:**
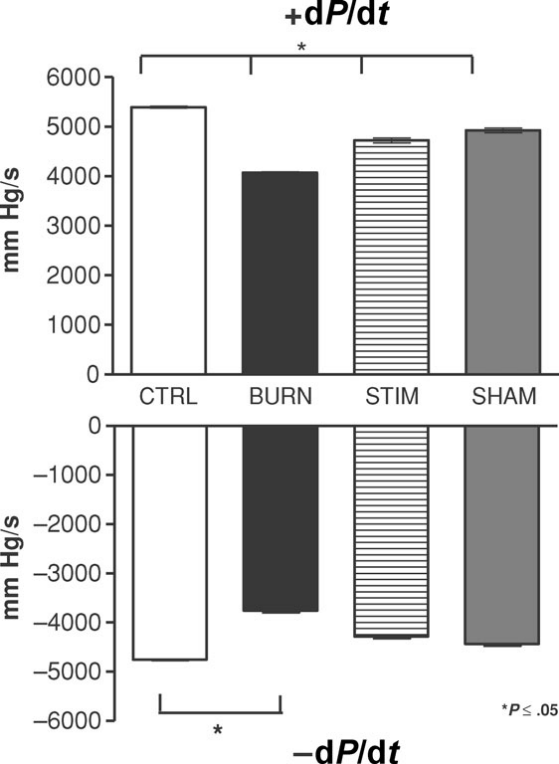
Intraventricular pressure rise and fall analysis (±d*P*/d*t*): Burned animals revealed decreased intraventricular pressure increments over time than nonburned controls and shams (+d*P*/d*t*, BURN vs CTRL and SHAM groups, *P* ≤ .05). Vagal electrostimulation induced significantly increased +d*P*/d*t* levels back to levels of sham and control animals (+d*P*/d*t*, STIM vs BURN group, *P* ≤ .05). The intraventricular pressure fall analysis (-d*P*/d*t*) showed a tendency toward normalized pressure fall values over time in animals with burn injury plus vagus nerve stimulation (-d*P*/d*t*, STIM group and BURN vs STIM group, *P* = NS). Burn injury resulted in a significantly slower pressure fall than measured in controls (-d*P*/d*t*, BURN vs CTRL, *P* ≤ .05).

Left ventricular EDP was analyzed in all experimental groups. Figure 3 shows that normal EDP values in the assessed control rats range around 18 mm Hg; conversely, in burned animals, decreased EDP values at about 11 mm Hg were found (Fig 2; CTRL vs BURN groups, *P* ≤ .05). Sham animals displayed decreased EDP levels compared with their control counterparts; burn injury and subsequent vagus nerve stimulation induced restoration of the EDP levels to sham levels but were not normalized to control animal levels after vagus nerve stimulation (Fig 2; SHAM vs CTRL groups [*P* ≤ .05]; STIM vs CTRL [*P* ≤ .05], and SHAM [*P* = NS] groups).

### Positive and negative pressure rise and fall

The intraventricular pressure rise analysis revealed that burned rats have significantly decreased intraventricular pressure increments over time than their nonburned control and sham counterparts (Fig 3; +d*P*/d*t*, BURN vs CTRL and SHAM groups, *P* ≤ .05). When burned animals received vagal electrostimulation, the +d*P*/d*t* levels increased significantly almost to the levels found in sham and control animals (Fig 3; +d*P*/d*t*, STIM vs BURN group, *P* ≤ .05). The intraventricular pressure fall analysis (-d*P*/d*t*) reflected the same pattern; however, animals with burn injury and vagus nerve stimulation (Fig 3; STIM group) showed a tendency toward normalized pressure fall values over time. But this effect was not statistically significant (Fig 3; -d*P*/d*t*, BURN vs STIM group, *P* = NS). Animals that underwent burn injury only displayed a significantly slower pressure fall than their noninjured control counterparts (Fig 3; -d*P*/d*t*, BURN vs CTRL, *P* ≤ .05).

### Cardiac contractility index

**Figure 4:**
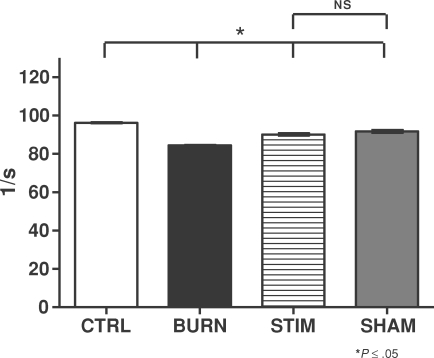
Cardiac contractility index (1/s). Controls had a higher contractility index than burned animals (CTRL vs BURN groups, *P* ≤ .05). Sham-injured animal index analysis revealed values in the control animal range (SHAM vs CTRL groups, *P* = NS). Burn injury plus vagus nerve stimulation restored index values to control and sham levels (STIM vs CTRL and SHAM, *P* = NS) and significant improvement was noted in vagus nerve–stimulated animals with burn trauma compared with burn-only animals (STIM vs BURN group, *P* ≤ .05).

The cardiac contractility index () was calculated as the maximal +d*P*/d*t* divided by the pressure at +d*P*/d*t*_max_. It is therefore expressed as per second (1/s). Our data showed that control animals had a higher contractility index than the burned animals (Fig 4; CTRL vs BURN groups, *P* ≤ .05). For sham-injured animals, the values for cardiac contractility index ranged around the levels found in control animals (Fig 4; SHAM vs CTRL groups, *P* = NS). Vagus nerve stimulation induced improvement of the index values back to the control and sham levels (Fig 4; STIM vs CTRL and SHAM, *P* = NS). The indices found in vagus nerve–stimulated animals that underwent burn injury were significantly improved compared with the burn injury–alone animals (Fig 4; STIM vs BURN group, *P* ≤ .05).

### Heart rate

We assessed the HR of each experimental animal before serum and tissue isolation. This was performed using left ventricular microcatheterization. There was no significant difference in the HRs of all study animals (data not shown).

## DISCUSSION

The parasympathetic, anti-inflammatory pathway (*inflammatory reflex*) can be activated by physical or pharmacological stimulation.^1^,^9^ In the present study, we used electrostimulation of the exposed vagus nerve and modified a technique that was previously described by Borovikova et al.^1^ The postburn inflammatory response includes cytokine and mediator generation and some of these induce cardiac dysfunction, either individually or in combination.^6^ On a molecular level, TNF-α, IL-1β, and IL-6 have been identified as primary myocardial depressant mediators in various experimental studies.^10^^-^12 We have shown previously that endotoxin (lipopolysaccharide) stimulation of cardiomyocytes isolated from burn-injured animals induces decreased sarcomere contractility.^13^ Adding on to this knowledge base, we as well as others have previously evaluated the potential of the parasympathetic system to attenuate these mechanisms.^7^,^14^ The background for this idea was the discovery of an anti-inflammatory pathway that is mediated by nicotinergic neurons and induced by acetylcholine as a neurotransmitter.^15^,^16^ This pathway has been termed the *inflammatory reflex* and has been shown to inhibit several inflammatory stress-related phenomena, for example, activation of the inflammatory reflex induced attenuation of TNF-α release after endotoxin challenge in an experimental endotoxin challenge model.^1^ Our own data reflecting the fact that vagus nerve stimulation after burn injury induced attenuation of proinflammatory mediator release prompted us to design this study.^7^ Linking the molecular event of proinflammatory cytokine release to cardiac function, we hypothesized that parasympathetic stimulation after experimental burn injury improves cardiac function as determined by in vivo microcatheterization of the left ventricle. Our data show that left ventricular pressure parameters, such as maximal, minimal, and mean, can be restored by vagus nerve stimulation after burn injury (Figs 1 and 2). Our results are in accordance with previous results published by Song et al^14^ showing that mean arterial pressure is significantly elevated after vagus nerve stimulation and concomitant burn injury. Also, in this context, it is noticeable that variations in the experimental setup seem to produce the same pattern of results. Song et al^14^ have used shorter duration of electrical pulses at 2 milliseconds, in contrast to our established setup that uses 40 milliseconds, but have also shown that in hemodynamic measurements such as mean arterial pressure measurements, the result of postburn vagus nerve stimulation is improvement in hemodynamic parameters. Adding on to this knowledge base, we have shown that EDP levels are significantly improved after burn injury and parasympathetic stimulation; however, in our experimental model, we consistently found no variations in HR in vagus nerve–stimulated animals, which is in contrast to the findings of Song et al.^14^ One of the potential reasons might be the longer electrical impulse duration used in our model (40 milliseconds compared with 2 milliseconds). Also, on the basis of the fact that vagal stimulation has been shown to induce variations in catecholamine release and acetylcholine availability, which has been reported by Kawada and colleagues^17^ in experimental cardiac ischemia models, one might speculate whether the catecholamine-induced tachycardia and vagus nerve stimulation–induced bradycardia may lead to balance and normocardia in our model. It is also interesting to note that burn injury did not have a significant influence on HR 3 hours postinjury, even though a tendency toward an increased HR in burned animals was noted in our experiments. Most important, vagus nerve stimulation of burned animals did not induce bradycardia or cardiac arrest. A systematic review of the current literature reflects that no such studies investigating the effect of parasympathetic stimulation on cardiac pacemaking elements in experimental models have yet been performed. Using our experimental model, which has been well established and evaluated in various experimental settings and designs, we could not reproduce the data published by Xia et al^18^ showing a decreased left ventricular EDP after experimental thermal injury. This may be due to differences in measurement techniques; we used in vivo left ventricular microcatheterization and did not use the intraventricular balloon technique, which is essentially an ex vivo technique and may thus reflect different EDP values than in vivo techniques.^18^

There is some evidence that the anti-inflammatory effect of vagus nerve stimulation in humans may be attenuated over time: In patients with epilepsy and associated inflammation, Barone et al^3^ reported that vagus nerve stimulation did not lead to decreased proinflammatory cytokine concentration after 3 months of vagus nerve stimulation. Although the number of assessed patients in their study is small, the interesting results should stimulate the expansion of clinical research efforts on the effects of vagus nerve stimulation in specific inflammatory situations in humans. These interesting findings should also stimulate the conduction of further studies designed to elucidate the molecular mechanisms of the inflammatory reflex in specific cell types and acute versus chronic underlying conditions (eg, systemic inflammatory response syndrome after burn trauma vs chronic inflammatory conditions). In this context, it is important to note that in sepsis, systemic inflammatory response syndrome, and multiorgan dysfunction syndrome, an autonomic cardiac dysfunction has been described that is clinically determined by attenuated sympathetic and parasympathetic mediated HR variability.^19^ Interestingly, our results revealed no significant alterations of the HR in animals of all experimental groups, specifically not in burn-only (BURN group) and burn plus vagus nerve stimulation (BURN + STIM) animals. One may speculate that this may be due to resuscitation and analgesia of the animals in the burn model; however, experiments without adequate resuscitation and analgesia to evaluate this effect are not possible for ethical reasons. In vitro experiments, for example, single-cell sarcomere contraction analysis (sarcomere shortening), should be conducted to evaluate the inflammatory reflex in infection. The synopsis of the various experimental setups in the field of burn trauma research also reflects the need for unification of experimental models, especially animal models, to improve comparability. The multifactorial pathogenesis of burn-induced organ dysfunction is well described: Other immunologic systems and factors, for example, the complement system, free radical, and nitric oxide (NO) generation, are centrally involved in the pathogenesis of postburn organ dysfunctions and immunological imbalances.^20^^-^22 Therefore, during the postburn phase, multiple immunologic mechanisms may overlap and each of them will likely contribute to the characteristic clinical and molecular postburn derangements.

In clinical settings, the inflammatory reflex was analyzed by Sher et al,^5^ who published data showing that nicotine had a positive effect in the clinical course of patients with chronic inflammatory bowel diseases. These data were also backed up by Guslandi and Tittobello,^23^ who showed that nicotine treatment induced longer remission phases in patients with chronic inflammatory bowel disease than did standard steroid treatment. One may also deduce from the fact that nicotine stimulation seems to also induce the inflammatory reflect that other parasympathomimetic substances may be able to incite the inflammatory reflex. This has been shown by Tracey,^24^ who demonstrated that the administration of CNI-1493, a tetravalent guanylhydrazone, attenuates the inflammatory response.

In summary, we believe to have provided experimental evidence that vagus nerve stimulation induces normalization of several cardiac function parameters after experimental thermal injury in our established model, which further undermines the theory that the blocking proinflammatory response after burn injury leads to improved organ function, as reflected by improved cardiac function in our study. The parasympathetic stimulation therefore seems to be an exiting potential strategy to normalize inflammatory imbalances after burn injury. This may lay ground for a novel therapeutic strategy, potentially using vagus nerve stimulators such as the ones recently approved by the Federal Drug Administration for the treatment of several neurologic diseases.^25^,^26^
